# Editorial: Neuronal Co-transmission

**DOI:** 10.3389/fncir.2019.00019

**Published:** 2019-03-26

**Authors:** John Apergis-Schoute, Geoffrey Burnstock, Michael P. Nusbaum, David Parker, Miguel A. Morales, Louis-Eric Trudeau, Erik Svensson

**Affiliations:** ^1^Department of Neuroscience, Psychology and Behaviour, University of Leicester, Leicester, United Kingdom; ^2^Department of Pharmacology and Therapeutics, The University of Melbourne, Parkville, VIC, Australia; ^3^Department of Neuroscience, Perelman School of Medicine, University of Pennsylvania, Philadelphia, PA, United States; ^4^Department of Physiology, Development and Neuroscience, University of Cambridge, Cambridge, United Kingdom; ^5^Instituto de Investigaciones Biomédicas, Universidad Nacional Autónoma de México, Mexico City, Mexico; ^6^CNS Research Group, Department of Pharmacology and Physiology, Department of Neurosciences, Faculty of Medicine, Université de Montréal, Montreal, QC, Canada; ^7^Department of Neuroscience, Functional Pharmacology, Uppsala University, Uppsala, Sweden

**Keywords:** neuropeptides, co-transmission, co-release, neurotransmitter segregation, neurotransmitter complexity

Neuronal co-transmission is now well-established as an aspect of nervous system function. However, this was not always the case, and acceptance of this important principle has required extensive work in a range of invertebrate and vertebrate systems (see Svensson et al. for a historical perspective). This work is reviewed in articles in this research topic, which we highlight in this Editorial.

A common principle highlighted by several articles in this research topic is that neuropeptides stored in dense core vesicles (DCVs) are co-localized with amines, and also with “classical” small molecule neurotransmitters stored in small synaptic vesicles in single terminals (SSVs; see Cropper et al.; Hökfelt et al.; Merighi; Nässel; Trudeau and El Mestikawy; Svensson et al.). SSVs can co-store and co-release protons and ATP, which can also elicit postsynaptic responses (Soto et al.; Svensson et al.). Soto et al. show that changes in extracellular pH activate acid sensing ion channels, and they discuss whether this is a synaptically-restricted signal or a volume modulator of neuron excitability.

Co-transmission in invertebrate neuronal circuits is highlighted by Nässel, Cropper et al., and Svensson et al.. Nässel summarizes the co-localization of neuropeptides and small molecule transmitters in *Drosophila* neuroendocrine cells, interneurons, and sensory and motor neurons, in circuits that influence learning and memory, circadian rhythms, and sensory, reproductive, developmental, and homeostatic functions. The circuit-related action of neuropeptides has been characterized in feeding circuits in the decapod crustacean stomatogastric ganglion (see the detailed discussion in Svensson et al.) and the *Aplysia californica* buccal ganglia (Cropper et al.). In *Aplysia*, analysis of the postsynaptic consequences of parallel neuropeptide actions has generated novel insights into the combinatorial effects of neuromodulators (Cropper et al.). Cropper et al. also address the role of different presynaptic activity patterns in the release of neuropeptides, an important aspect required for understanding the endogenous release of co-localized transmitters. Neuronal co-transmission has also been studied in vertebrate spinal circuits, both in dorsal horn sensory pathways and in spinal circuits involved in locomotion (see Svensson et al.).

The mammalian peripheral nervous system has been an important preparation for studying co-transmission in relation to autonomic responses, and it provided some of the key early physiological evidence for co-localization (Burnstock, 1976; see Svensson et al.). For example, in sympathetic neurons in the rat superior cervical ganglion acetylcholine and GABA are found in the same neurons, albeit segregated into different axonal varicosities (this has been defined as co-existence rather than co-localization; the latter reflects two or more transmitters contained in a single terminal; Merighi, 2002). Other sympathetic neurons co-localize noradrenaline and ATP (see Svensson et al. and references therein). Merino-Jiménez et al. show that sympathetic neurons of the rat superior cervical ganglion segregate their neurotransmitters and co-transmitters to separate varicosities of single axons. They discuss whether sympathetic dysfunction in stress and hypertension correlate with changes in segregation, addressing the important question of the plasticity of transmitter systems during different functional states (Merino-Jimenez et al.).

Co-localization and co-existence occurs in multiple areas in the mammalian central nervous system, many of which are of clinical interest. For example, dopaminergic neurons in the ventral tegmental area contain glutamate and neuropeptides and project to both the nucleus accumbens and the neocortex (Pérez-López et al.; Trudeau and El Mestikawy.). In the nucleus accumbens, dopamine and glutamate, which are segregated to different varicosities, may play a role in drug addiction (Papathanou et al.). Dopamine and GABA released from ventral tegmental neurons can also influence the excitability of the prefrontal cortex (Pérez-López et al.). Dopaminergic projections from the substantia nigra and ventral tegmental area co-localize dopamine and GABA and make synapses on medial spiny neurons in the striatum. These dopamine neurons degenerate in Parkinson's disease, and understanding co-transmission from these neurons could provide novel insight into the treatment of Parkinson's disease symptoms (see Shen et al., 2018; Steinkellner et al., 2018). Trudeau and El Mestikawy specifically address the extent and functions of glutamate co-transmission in different classes of central neurons. They highlight the heterogeneous nature of glutamate co-transmission in different brain regions, and its implications for various functional processes like spatial learning, drug abuse and mood regulation. Hökfelt et al. discuss the co-localization of the neuropeptide galanin in noradrenergic neurons in the locus coeruleus. Galanin transmission is enhanced by stress and they suggest this could contribute to the induction of major depressive disorders. Co-localization of the neuropeptide orexin/hypocretin with glutamate helps regulate sleep/awake cycles. During waking, they are co-released: glutamate evokes fast excitation of histaminergic neurons and orexin/hypocretin boosts and prolongs this response by evoking a slow excitation. Dysfunction of these neurons causes narcolepsy: it is thus important to understand the functional role of this co-transmission in maintaining wakefulness (Svensson et al.).

Neuronal co-transmission has thus been extensively studied. However, there are many open questions, several of which are highlighted in this research topic. For example, we lack detailed knowledge about the subcellular organization of co-localized neurotransmitters and the presynaptic signals that differentiate their release. Merighi reviews the molecular composition and mechanisms of filling and release of large DCVs. He highlights future directions of research in the synthesis and storage of multiple transmitters in DCVs, including the possibility of selective transmitter sorting to different processes (see for example Fortin et al., 2019). An additional complexity is the combinatorial effects resulting from parallel neurotransmitter actions that can give rise to non-additive effects. This presents a challenge for any pharmacological intervention in the nervous system, as drug effects will depend on the background state of the system. Determining the general principles of these complex actions is thus a pressing need. New methodological developments that combine electrophysiology with optogenetic activation/inhibition of specific neuronal populations has started to provide insight into the functional roles and co-release of co-localized neurotransmitters (Papathanou et al.; Pérez-López et al.; Svensson et al.). The use of genetically modified animals (see Nässel; Svensson et al.) is also likely to provide important insights into the functional significance of co-transmission.

## Author Contributions

All authors listed have made a substantial, direct and intellectual contribution to the work, and approved it for publication.

### Conflict of Interest Statement

The authors declare that the research was conducted in the absence of any commercial or financial relationships that could be construed as a potential conflict of interest.
